# The Number of Pregnancies and Deliveries and Their Association with Selected Morphological and Hemodynamic Parameters of the Pelvic and Abdominal Venous System

**DOI:** 10.3390/jcm10040736

**Published:** 2021-02-12

**Authors:** Cezary Szary, Justyna Wilczko, Dominika Plucinska, Anna Pachuta, Marcin Napierala, Anna Bodziony, Michal Zawadzki, Tomasz Grzela

**Affiliations:** 1 Clinic of Phlebology, 02-034 Warsaw, Poland; cezary.szary@klinikaflebologii.pl (C.S.); justyna.wilczko@klinikaflebologii.pl (J.W.); dominika.plucinska@klinikaflebologii.pl (D.P.); anna.pachuta@klinikaflebologii.pl (A.P.); marcin.napierala@klinikaflebologii.pl (M.N.); anna.bodziony@klinikaflebologii.pl (A.B.); michal.zawadzki@klinikaflebologii.pl (M.Z.); 2 Diagnostic Imaging Center MRI & CT, Center of Sport Medicine, 02-034 Warsaw, Poland; 3 Department of Radiology, Center of Postgraduate Medical Education, 01-813 Warsaw, Poland; 4 Department of Histology and Embryology, Medical University of Warsaw, 02-004 Warsaw, Poland

**Keywords:** chronic venous disease, delivery, ovarian veins, parauterine veins, pelvic veins insufficiency, pregnancy, venous abnormalities

## Abstract

Background: Although pregnancy has been identified as one of the risk factors for venous disease, the mechanism of this interaction remains unclear. Possibly, pregnancy results in overstrain and vein dilatation, which exceed their durability and persist after pregnancy. The aim of this study was the assessment of the relationship between the number of pregnancies in women with venous disease and the selected parameters of their venous systems. Patients and methods: The retrospective assessment concerned 518 patients subjected to the diagnostics of the venous system in the lower limbs and the abdomen/pelvis using ultrasound scan and magnetic resonance or computed tomography. Results: We found that the occurrence of pelvic venous symptoms increases proportionally to the number of pregnancies and is correlated with ovarian and parauterine vein dilatation/incompetence (e.g., 13.5% of nulliparous women reported pelvic pain, and reflux in left ovarian veins was detected in 21.4% of the patients from that group, whereas in women after two pregnancies, pain and reflux concerned 22.8% and 90.6% of patients, respectively). In the nulliparous group, the development of venous disease resulted from the presence of anatomic abnormalities in abdominal/pelvic veins. Conclusions: Our report proved that the number of pregnancies is correlated with the incidence of pelvic vein insufficiency. Although not specifically addressed in this study, some correlation was found with saphenous disease as well. However, further studies are necessary to provide more evidence about the role of pelvic vein insufficiency in chronic venous disease of the lower limbs.

## 1. Introduction

Venous insufficiency is a common clinical problem, although its prevalence varies among different statistics. When assessed as a fully symptomatic disease, it may affect approximately 60–65% individuals in the adult population [1,2,3]. However, if the early stage is also included, it may affect even 83% of the adult population [4,5]. The etiopathogenesis of chronic venous disease (CVD) is highly complex. Usually, it is considered as the result of cooperation between several external and intrinsic/inborn risk factors [6,7]. The first include low physical activity, a sedentary lifestyle, and obesity [7,8]. The intrinsic risk factors are defined mainly by a patient’s genetics and comprise various conditions. Some of them, such as female gender and sex-related features (including sex hormones), or thrombophilia, are well identified. Others, among them the inborn abnormalities of the venous system, with May–Thurner syndrome, combined venous malformations (e.g., Klippel–Trenaunay syndrome), or weakness and fragility of the venous wall due to the polymorphism of collagen-encoding genes, remain less recognized [6,9,10,11]. Besides the aforementioned, presumably the most important factor for the development of venous disease is pregnancy, or more precisely, multiparity [3,11,12,13].

Interestingly, although numerous studies have proved the number of pregnancies to positively correlate with the development of lower limb venous insufficiency, the detailed mechanism responsible for such a relationship remains unclear. It has been found that the risk of CVD changes from 20% in nulliparous to 41% in primiparous women, and further increases with each next pregnancy [12,14]. Presumably, it may be due to the overload of the venous system due to its compression by gravid uterus in late pregnancy [12,15,16]. If this congestion exceeds the compensation/regenerative potential of the venous wall, it may result in its irreversible damage, leading to permanent dilatation and venous insufficiency.

According to several reports, the hemodynamic and morphological alterations during pregnancy concern the appearance and worsening of varicose veins in the lower limbs and in the vulvo-perineal region [12]. Noticeably, the most significant impairment in blood flow was observed locally, in pelvic and abdominal venous circulation [16]. Thus, since the veins of these locations are subjected to extraordinary overstrain during pregnancy, it is plausible that some morphological and hemodynamic changes could persist after pregnancy, too. However, sufficient data to confirm this hypothesis have not been available so far. Therefore, the aim of our study was the assessment of the selected morphological and hemodynamic parameters of the pelvic and abdominal venous system with regard to the number of pregnancies and deliveries experienced by women with venous disease. The possible association of the mentioned parameters with the occurrence of miscarriages in that group was analyzed.

## 2. Patients and Methods

Aretrospective analysis was performed using data collected from the database of our clinic for the years 2017–2019. The data originated from 2136 records, corresponding to consecutive patients subjected to routine diagnostic procedures in our clinical practice. The concept of the study was reviewed and formally approved by the Local Ethics Committee at the Medical University of Warsaw (decision No. AKBE/181/2020).

The records were collected based on the standard protocol of our clinic. At the beginning, patients were asked to answer several questions from a standardized questionnaire, concerning patient demography, current symptoms and general health status, pregnancies, deliveries, concomitant diseases, and previous treatments. Then, the patients were subjected to a routine two-step diagnostic algorithm. In the first step, the patients were subjected to an ultrasound examination of their venous system using Toshiba Xario 100 (TOSHIBA/Canon Medical Systems Co., Otawara, Tochigi, Japan) with a linear probe (8–14 MHz) for the assessment of lower limb veins, and a convex probe (6–9 MHz) for the examination of abdominal and pelvic veins.

The limb veins’ assessment was performed in the standing position, whereas abdominal and pelvic veins were scanned in the semi-sitting and lying (supine) position. The color duplex ultrasound (CDU) examination focused on the detection and measurement of reflux, defined as the reversed flow >500 ms, spontaneous or induced by distal compression or Valsalva maneuver, as well as the identification of reflux pathways in the superficial venous system of the lower limbs.

The transabdominal CDU scan concerned the morphology and blood flow in large venous trunks, including the inferior vena cava (IVC) and iliac veins—common, external, and internal (CILV, EILV, and IILV, respectively). Then, the diameter and blood flow were assessed in both renal veins (left and right—LRV and RRV, respectively), both ovarian veins (left and right—LOV and ROV, respectively), as well as the parauterine veins (PUV).

The patients with any detected (or suspected) significant abnormalities were subjected to further detailed examination using either magnetic resonance venography (MR-V) or computed tomography venography (CT-V). The first was the method preferred in patients with unstable hyperthyroidism or known allergy to iodine and in women of childbearing potential. The protocols of image acquisition and processing were optimized to produce compatible data from both methods, as verified in three independent patients.

The examination was performed using an intravenous contrasting agent, injected withan automated syringe at the flow rate of 3.5–4 mL/s. The scanning area concerned the venous system—from IVC connection with the right atrium on the top, to the proximal one-third of the thigh on the bottom. The assessment included the verification of previous findings from ultrasound examinations and further extended the evaluation of possible vein abnormalities. The measurement of selected parameters was done according to standardized protocol—always in the same phase after contrasting agent infusion, in the same location (or part of the vein) and in the plane perpendicular to the vein axis.

MR-V examination was performed using an Ingenia 3.0T MR scanner (Philips, Netherlands). Initial imaging was done without contrast enhancement, using morphological sequences—T2, FatSat T2, and balanced turbo field echo (BTFE) gradient sequence. Next, after injection of the gadolinium contrasting solution (ProHance), images were acquired in dynamic sequences with up to sixcontrasting phases. Finally, delayed contrast enhancement with mDIXON high-resolution sequence was used for the imaging of pelvic and vulvoperineal veins. CT-V imaging was performed using a 128-multislice, 64-row Incisive CT scanner (Philips, Netherlands). The examination protocol concerned two venous phases—the early, 50 s from achieving the saturation peak of the contrast agent (Ultravist or Iomeron) in the abdominal aorta, and the late, after 120 s.

The results from the aforementioned examinations were stored in a clinical database, to be used as the basis for decisions regarding further treatment of patients. For the present study, the database was searched using the following inclusion criteria: female, clinical symptoms of venous disease—C1 to C4 (according to the classification concerning clinical symptoms, etiology, anatomy, and pathophysiology (CEAP)) [17], available complete data from extended CVD examination with either MR-V or CT-V, no active malignancies, and no active thrombosis.

All selected inclusion criteria were met in 518 from the initial 2136 records, which were then extracted from the database, anonymized, and used for analysis. The randomly selected 25 records from the extracted group were subjected to the verification of their accuracy by direct comparison with the source data in clinical documentation. The extracted records were further filtered and sorted using additional differentiating factors: “number of pregnancies” and “number of deliveries.” Selected morphological parameters of the venous system were then compared among these subgroups with regard to the number of pregnancies, or the number of deliveries, as a stratifying factor.

Finally, both data sets were analyzed using either descriptive statistics or comparative assessment, with the Mann–Whitney U test, at *p* < 0.05 being considered statistically significant. With regard to the selected parameters, their association with the patients’ clinical status was evaluated using either odds ratio (OR) or risk ratio (RR) with 95% confidence interval (95% CI).

## 3. Results 

Based on the number of pregnancies, the 518 patients were allocated to respective subgroups. Approximately 24.3% of them were found to be nulliparous (P0), whereas 19.1% of the women experienced one pregnancy (P1), and 33.0%two pregnancies (P2). The remaining 23.5% comprised patients after three (P3 with 15.0%) or more pregnancies (P4+ with 8.5%). As was expected, the women from the P0 subgroup were statistically significantly younger compared to others. Therefore, to reduce age-related bias in further assessment, an additional age-matched subgroup (P0^AM^) was created by extracting 80 women at age above 30 from the P0 group.

Although all patients selected for the analysis suffered from venous insufficiency (as it was actually the main inclusion criterion), less than half of them reported pain or discomfort concerning their legs, except for the P4+ subgroup, where these symptoms were experienced by 70.4% women. The calculated odds ratio for the occurrence of leg pain/discomfort in this group, compared to P0, was OR = 3.3, with 95% CI = 1.6–6.8, at *p* = 0.001. Noticeably, chronic pain/discomfort in the lower parts of the abdomen or pelvic region was reported by 20.1% patients from the whole group. However, it concerned only 13.5% women in the P0 and 15.1% in the P1 subgroups. By contrast, approximately one-fourth of the individuals in the P3 and one-third in the P4+ subgroups experienced chronic pelvic pain (CPP). For both subgroups, the calculated odds ratios for the presence of CPP in these groups, compared to P0, were OR_(P3)_ = 2.1, with 95% CI = 1.0–4.3, at *p* = 0.05, and OR_(P4)_ = 3.9, with 95% CI = 1.7–9.4, at *p* = 0.002.

More than one-third of the women from the whole assessed group were previously subjected to the treatment of varicose veins of the lower limbs. It is noteworthy that, when analyzed in subgroups, except for only 22.2% in the nulliparous group, approximately 40% women from each of the remaining subgroups had already undergone varicose vein treatment. Some of them, especially from the P3 and P4+ subgroups, were treated with more than a single approach, and using more than one method. Unexpectedly, despite previous treatment, 93.6% of the patients revealed reflux in lower limb superficial veins. Although some trend in the occurrence and extension of venous reflux was observed with regard to the number of pregnancies, this association did not reach statistical significance. Similarly, despite a clear tendency to do so, the abovementioned ultrasound findings did not reveal a statistically significant correlation with the occurrence of subjective leg symptoms. The clinical characteristics of the analyzed subgroups are summarized in Table 1.

**Figure 1:**
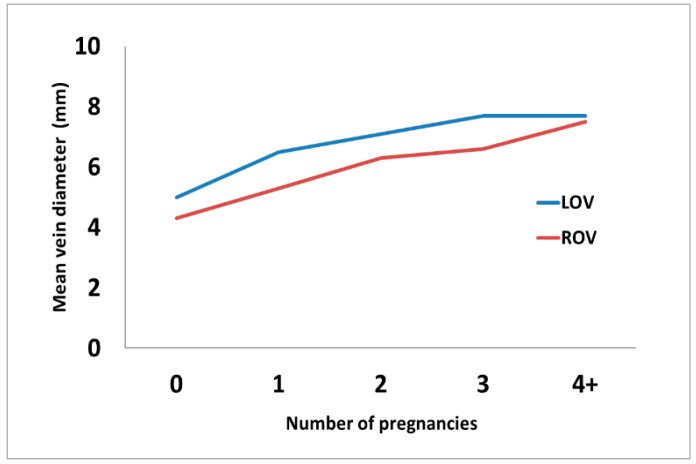
The graph shows the mean diameter (in mm, vertical axis) of both ovarian veins with regard to the number of pregnancies (horizontal axis). Abbreviations: LOV: left ovarian vein, ROV: right ovarian vein, and “4+”: 4 or more pregnancies.

The pregnancy-based subgroups were further analyzed with regard to the results of the extended imaging of the abdominopelvic venous system using MR-V or CT-V. It was found that the mean diameters of both ovarian veins gradually increase after each pregnancy and are significantly higher, compared to the P0 subgroup, in all the remaining subgroups, as shown in Figure 1 and Table 2.

Noticeably, the increasing number of pregnancies was accompanied by the increasing frequency of ovarian veins, with the reflux detected either at CDU examination or with extended imaging. The calculated risk ratio (RR) for the occurrence of reflux in LOV after the first pregnancy was RR = 2.21, with 95% CI = 1.49–3.28, at *p* = 0.0001, and it further increased after the second and further pregnancies (RR = 4.16, 95% CI = 2.97–5.82, at *p* < 0.0001). The increase in the reflux occurrence was also observed in ROV; however, it reached statistical significance only after the second and further pregnancies (RR = 12.21, 95% CI = 5.13–29.06, at *p* < 0.0001), as shown in Table 2.

Unexpectedly, the extended assessment in MR-V or CT-V showed that, apart from the dilatation of ovarian veins, approximately one-third of all patients reveal several clinically relevant morphological variants or abnormalities in their venous system. The majority of them (almost 40%) concerned the left renal vein/left ovarian vein axis (LRV/LOV), although in the P1 subgroup, they contributed to 62.9% of the observed alterations. The second group of abnormalities, in the order of their prevalence (approx. 30%), concerned morphological and functional impairments of iliac veins. They included the compression or hypoplasia of the left common iliac vein (LCILV), also known as May–Thurner syndrome, or the variants and confluence impairments of internal iliac veins (IILV) to the CILV, e.g., left-to-right or right-to-left transpositions. Interestingly, in the P4+ subgroup, various alterations in the venous system were observed in nearly half of the patients. Noticeably, the observed high frequency of abnormalities in that subgroup, besides those concerning either ovarian or iliac vein axes, were related toother changes, including postoperative or post-thrombotic ones. The aforementioned findings in relation to the number of pregnancies are summarized in Table 2.

**Figure 2:**
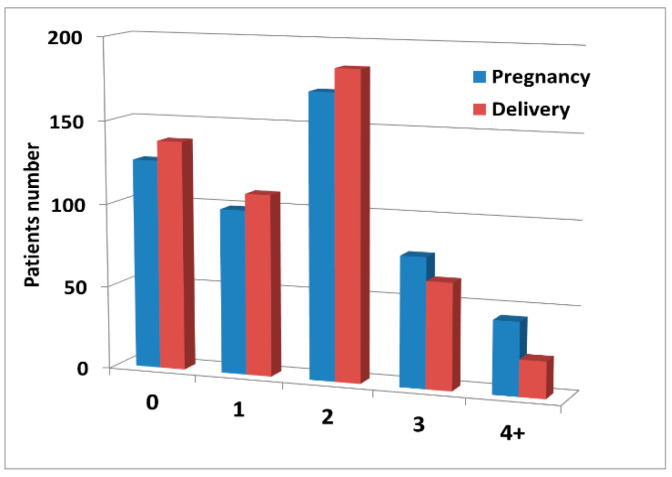
The graph shows the number of patients (vertical axis) corresponding to the number of pregnancies (blue bars) or the number of deliveries (red bars) per individual, respectively (horizontal axis). “4+”: 4 or more. Note that the bars corresponding to the number of deliveries differ from the respective “Pregnancy” bars due to miscarriages.

The influence of the number of deliveries on the development of morphological alterations in the abdominopelvic venous system was assessed in a way similar to that described previously for pregnancies. Using the number of deliveries as a stratifying factor resulted in patients’ allocation to groups with the numbers of individuals different from those in pregnancy groups, due to 119 miscarriages experienced by 79 women. The distribution of the number of pregnancies and deliveries is shown in Figure 2.

The gradual increase of diameters in both ovarian veins correlated with the number of deliveries and this trend was very similar to that observed in pregnancy. The summary of these observations is shown in Table 3.

**Figure 3:**
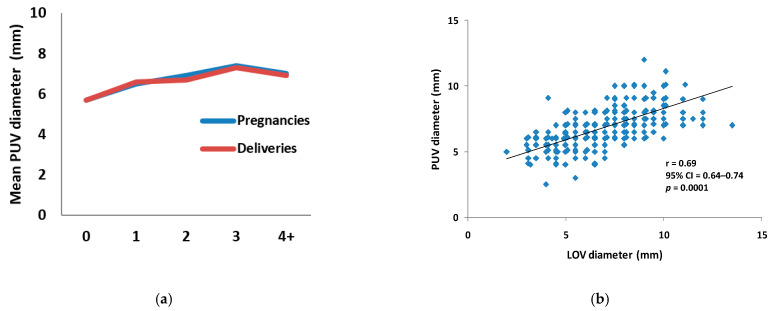
Mean parauterine vein diameter. (**a**) The graph shows the mean diameters (in mm, vertical axis) of parauterine veins with regard to the number of pregnancies or deliveries, respectively (horizontal axis). (**b**) The graph shows the relationship between the diameters of left ovarian and parauterine veins. Each dot represents the respective measurements from a single individual. Abbreviations: LOV: left ovarian vein, PUV: parauterine veins, and “4+”: 4 or more.

The increasing numbers of both pregnancies and deliveries resulted also in the enlargement of the mean diameters of parauterine veins (Figure 3a, Table 1 and Table 2). It is noteworthy that this observation was consistent with the results of the statistical analysis, which confirmed the statistically significant correlation between the diameters of the LOV and PUV, with the calculated correlation coefficient *r* = 0.69, 95% CI = 0.64–0.74, at *p* = 0.0001 (Figure 3b).

The results of extended diagnostics of the abdomino-pelvic venous system, especially the presence of morphological and functional anomalies, were also assessed for their presumable association with the occurrence of miscarriages in the analyzed group. It was found that the occurrence of anomalies in the whole group did not differ significantly from that in women who experienced miscarriages, and was 32.2% vs. 30.4%, respectively. Although the frequency of those abnormalities was markedly increased in women who lost all pregnancies (47.6%), this difference, when compared to miscarriages in women without venous anomalies, appeared not significant. Assessment of the diameters of ovarian and parauterine veins showed that the mean size of those veins was statistically significantly lower in patients with miscarriages, especially in women who lost all pregnancies, compared to those without miscarriages. The short summary of this assessment is shown in Table 4.

## 4. Discussion

Although the role of pregnancy as a risk factor in the development of chronic venous disease has been postulated by many authors [3,11,14], the mechanism that can explain this relationship remains unclear. Since most studies focused on the impairment of the lower limb venous system only, little was known about the pregnancy-induced malfunction of abdominal and pelvic veins [12,16]. Our report provides some data and may add some explanation of that phenomenon. Moreover, it supports and extends the findings from previous studies, suggesting a possible link between the presence of varices in the lower limbs and the abdominal and/or pelvic vein insufficiency (PVI) [18,19]. It is noteworthy that literature reports that the signs and symptoms related to pelvic vein congestion and/or insufficiency, among them chronic pelvic pain/discomfort, excessive or prolonged menorrhagia, and hemorrhoids, were observed in 20–40% patients with CVD [20]. In our study, CPP was reported by 20.1% women in the whole group. Nevertheless, when assessed in subgroups, this symptom was experienced by 13.5% patients in the nulliparous group (P0), but its occurrence gradually increased after each pregnancy, up to 29.5% in women after four or more pregnancies (P4+).

The link between pregnancy, PVI, and the manifestation of varicose veins in the lower limbs has been emphasized in a few publications concerning recurrent varicose veins [21,22,23,24,25]. Venographic studies using either computed tomography or magnetic resonance imaging play a key role in that research. Dynamic venography not only allows detailed morphological and hemodynamic assessment of ovarian reflux but also enables the precise identification of leakage points, which transfer the reflux from pelvic veins to the superficial venous system of the lower limb [26]. Considering this, a few authors have proposed scleroembolization of pelvic veins in selective cases of varices in the lower limbs [18,19]. These cumulative data reinforce the need to take into consideration also the likelihood of PVI in the diagnostic and therapeutic approach to lower limb varicose veins [18,19,21].

Although significant morphological and functional alterations in abdominal and pelvic veins during late pregnancy have already been reported by several authors, it is believed that the vast majority of those cases are fully reversible after delivery [15,16,25]. However, our observations suggest that the compensation potential of overstrained abdominal and pelvic veins may be sufficient in half of the women until the second pregnancy only, whereas the second and each next pregnancy leads to the irreversible failure of the LRV/LOV axis in nearly 90% women. The exceptional role of this morphological and functional venous unit, designated as the LRV/LOV axis, in the pathophysiology of PVI results from the specific anatomical and hemodynamic relation between LRV, LOV, and other abdominal and pelvic vessels [23]. The mean diameters of LOV and PUV increased significantly at the first and each subsequent pregnancy. Moreover, both variables revealed a strong, statistically significant correlation.

Since it is anatomically and functionally distinct from LOV, the impairment of the ROV axis, apart from some relatively rare cases, appears later, usually after the second pregnancy, and seems to be secondary to the dysfunction of LRV/LOV and PUV. This conclusion is based on the observation that, although the significant ROV dilatation was observed already after first pregnancy, it was initially related to overload rather than insufficiency; furthermore, less than 10% patients from P1 reveal reflux in ROV, as found in CDU assessment or with extended imaging with MR-V or CT-V. Noticeably, the reflux in ROV was detected in approximately half of the patients after the second or further pregnancies, while LOV insufficiency in the same groups concerned almost 90% of the individuals.

The redirection of excessive blood from the LRV/LOV axis to parauterine veins may occur as the effect of simple pregnancy-induced PVI. It may also be a consequence of impaired outflow due to some inborn venous anomalies. The latter include various anatomical abnormalities of LRV (mainly its entrapment between the aorta and the superior mesenteric artery, also known as the “nutcracker” phenomenon, or LRV compression due to its retroaortic location), as well as some LOV anomalies, usually concerning atypical blood drainage (into accessory renal veins, or even independent of LRV) [21]. Each of the aforementioned factors, as well as some other venous irregularities (e.g., concerning iliac veins), may be sufficient to overload ovarian and/or parauterine veins. Then, the overstrained PUV may further propagate the reflux through perineal connections—so-called leakage points—to the lower limb venous system [19,21,24].

The pathomechanisms described above provide an explanation for the role of pregnancy in the development of PVI. The increased number of pregnancies indisputably has a detrimental effect on the abdominal and pelvic venous system, the progression of PVI, and, presumably, the damage of the venous system in the lower limbs. Noticeably, the assessed morphological parameters (LOV, ROV, and PUV diameters) in women with miscarriages were similar to the respective data in the nulliparous group (P0). Hence, since early miscarriages did not significantly affect the condition of abdominal and/or pelvic veins, this observation may confirm the predominant role of mechanic/hemodynamic overstrains due to vein compression by the enlarged uterus in late pregnancy [16].

Dysfunction of pelvic veins may apply to nulliparous women as well. However, in their case, instead of pregnancy, the impairment of the LRV/LOV axis may result from the presence of various clinically relevant venous abnormalities [27]. In fact, one-fourth of the patients in the P0 subgroup revealed the features of PVI, including reflux in ovarian veins, which in all analyzed cases was due to the presence of various abnormalities, involving mainly the LRV/LOV axis or iliac veins.

Since the above-mentioned venous anomalies play a key role in the development of PVI, it is plausible that they can also increase the risk of miscarriages [28]. Indeed, the frequency of such alterations in women who experienced miscarriages was markedly increased compared to patients without miscarriages. However, possibly due to the small group of women experiencing the miscarriages of all their pregnancies, this difference appeared statistically nonsignificant.

In summary, our report provides the evidence that the number of pregnancies correlates with the increasing impairment of either morphological and functional or the abdominal and pelvic venous system in women with chronic venous disease. Although not specifically addressed in this study, some correlation was found with lower limb venous disease as well. Further prospective studies, involving patients from all CEAP classes, are necessary to provide more evidence about the role of PVI in the occurrence of varicose veins or of chronic venous disease of the lower limbs.

## Figures and Tables

**Table 1:** Clinical characteristics of the cohort patients with respect to the number of pregnancies (P).

Parameter or Variable	Whole Group	P0	P0^AM^	P1	P2	P3	P4+
Cases in group	518	17 (13.5%)	81 (15.6%)	99 (19.1%)	171 (33.0%)	78 (15.0%)	44 (8.5%)
Age	42.3 ± 10.5 *	36.5 ± 11.6	42.2 ± 11.1	42.2 ± 9.5	44.4 ± 9.4	44.5 ± 8.7	46.8 ± 9.9
Pregnancies	1.7 ± 1.4	126 (24.3%)	0	1	2	3	4.9 ± 1.5
Deliveries	1.5 ± 1.2	0	0	0.9 ± 0.3	1.9 ± 0.4	2.6 ± 0.5	3.5 ± 1.6
Leg symptoms	253 (48.8%)	0	41 (50.6%)	49 (49.5%)	77 (45.0%)	38 (48.7%)	31 (70.4%) *
CPP	104 (20.1%) *	55 (43.6%)	14 (17.3%)	15 (15.1%)	39 (22.8%) *	19 (24.3%) *	13 (29.5%) *
Reflux in GSV/SSV:	485 (93.6%) *	101 (80.2%)	71 (87.6%)	95 (95.9%) *	168 (98.2%) *	77 (98.7%) *	44 (100.0%) *
—Axial (with SFJ)	123 (23.7%) *	21 (16.7%)	15 (18.5%)	24 (24.2%) *	42 (24.6%) *	23 (29.5%) *	13 (29.5%) *
—Partial (non-axial)	362 (69.9%) *	80 (63.5%)	56 (69.1%) *	71 (71.7%) *	126 (73.7%) *	54 (69.2%) *	31 (70.5%) *
—Both legs affected	52 (10.0%) *	7 (5.6%)	5 (5.6%)	11 (11.1%) *	17 (9.9%) *	8 (10.3%) *	9 (20.5%) *
PTS alterations	18 (3.5%) *	2 (1.6%)	1 (1.6%)	3 (3.0%) *	7 (4.1%) *	1 (1.3%)	5 (11.4%) *
VV treatment	183 (35.3%) *	28 (22.2%)	20 (24.7%)	41 (41.4%) *	65 (38.0%) *	32 (41.0%) *	17 (38.6%) *
—Surgery	104 (20.1%)	11 (8.7%)	9 (11.1%)	22 (22.2%)	43 (25.1%)	14 (17.9%)	14 (31.8%)
—Thermoablation	41 (7.9%)	7 (5.5%)	2 (2.5%)	13 (13.1%)	14 (8.2%)	4 (5.1%)	3 (6.8%)
—Sclerotherapy	89 (17.2%)	13 (10.3%)	12 (14.8%)	26 (26.3%)	32 (18.7%)	15 (19.2%)	3 (6.8%)

The values shown in the table represent mean ±SD, or the number (percent) of cases in the respective group. Abbreviations: SD: standard deviation, CPP: chronic pelvic pain, GSV: great saphenous vein, SSV: small saphenous vein, SFJ: saphenofemoral junction, PTS: post-thrombotic syndrome, and VV: varicose veins. The values marked with asterisks (*) are significantly different compared to the corresponding values in the P0^AM^ group.

**Table 2:** Association of selected morphological parameters of abdominal/pelvic veins with the number of pregnancies (P).

Parameter or Variable	Whole Group	P0	P0^AM^	P1	P2	P3	P4+
Cases in group	518	126 (24.3%)	81 (15.6%)	99 (19.1%)	171 (33.0%)	78 (15.0%)	44 (8.5%)
LOV diameter (mm)	6.7 ± 2.0 *	5.0 ± 2.0	5.1 ± 1.6	6.5 ± 1.9 *	7.1 ± 1.7 *	7.7 ± 1.8 *	7.7 ± 1.9 *
ROV diameter (mm)	5.9 ± 1.8 *	4.3 ± 1.0	4.2 ± 0.8	5.3 ± 1.4 *	6.3 ± 1.8 *	6.6 ± 1.6 *	7.5 ±2.2 *
PUV diameter (mm)	6.6 ± 1.4 *	5.7 ± 1.1	5.7 ± 1.1	6.5 ± 1.5 *	6.9 ± 1.3 *	7.4 ± 1.2 *	7.0 ± 1.3 *
Reflux in LOV	335 (64.7%) *	27 (21.4%)	21 (25.9%)	47 (47.5%) *	155 (90.6%) *	67 (85.9%) *	39 (88.6%) *
Reflux in ROV	156 (30.1%) *	5 (4.0%)	3 (3.7%)	9 (9.1%)	74 (43.3%) *	43 (55.1%) *	25 (56.8%) *
Morphological variants							
or abnormalities:	167 (32.2%)	43 (34.1%)	29 (35.8%)	27 (27.3%)	52 (30.4%)	25 (32.0%)	20 (45.4%)
—Concerning LRV/LOV	65 (38.9%)	18 (41.9%)	16 (55.2%)	17 (62.9%)	16 (30.8%)	10 (40.0%)	4 (20.0%)
—Concerning RRV/ROV	27 (16.2%)	5 (11.6%)	2 (6.9%)	2 (7.4%)	8 (15.4%)	5 (20.0%)	7 (35.0%)
—Concerning ILV	51 (30.5%)	14 (32.5%)	8 (27.6%)	6 (22.2%)	19 (36.5%)	6 (24.0%)	6 (30.0%)
—Concerning IVC	15 (9.0%)	5 (11.6%)	2 (6.9%)	1 (3.7%)	6 (11.5%)	3 (12.0%)	0 (0%)
—Others	9 (5.4%)	1 (2.3%)	1 (3.4%)	1 (3.7%)	3 (5.8%)	1 (4.0%)	3 (15.0%)

The values shown in the table represent mean ±SD, as well asthe number (percent) of cases within the respective group. Abbreviations: SD: standard deviation, LOV: left ovarian vein, ROV: right ovarian vein, PUV: parauterine vein, LRV: left renal vein, RRV: right renal vein, ILV: iliac veins, and IVC: inferior vena cava. The values marked with asterisks (*) were statistically significantly different compared to corresponding values in the P0^AM^ group.

**Table 3:** Association of selected morphological parameters of abdominal/pelvic veins with the number of deliveries (D).

Parameter or Variable	Whole Group	D0	D1	D2	D3	D4+
Cases in group	518	138 (26.6%)	109 (21.0%)	185 (35.7%)	64 (12.3%)	22 (4.2%)
LOV diameter (mm)	6.7 ± 2.0 *	5.0 ± 1.6	6.6 ± 1.9 *	7.1 ± 1.8 *	7.8 ± 1.7 *	7.8 ± 1.5 *
ROV diameter (mm)	5.9 ± 1.8 *	4.3 ± 1.0	5.4 ± 1.4 *	6.5 ± 1.7 *	7.1 ± 1.8 *	7.6 ± 2.4 *
PUV diameter (mm)	6.6 ± 1.4 *	5.7 ± 1.0	6.6 ± 1.5 *	6.7 ± 1.3 *	7.3 ± 1.2 *	6.9 ± 1.4 *

The values shown in the table represent mean ±SD, as well asthe number (percent) of cases within the respective group. Abbreviations: SD: standard deviation, LOV: left ovarian vein, ROV: right ovarian vein, PUV: parauterine vein. The values marked with asterisks (*) were statistically significantly different compared to the corresponding values in the D0 group.

**Table 4:** Association of selected morphological parameters of abdominal/pelvic veins with the number of miscarriages (M) in the respective pregnancy group (P).

Parameter/Group	Number of Variants or Anomalies	Number of Miscarriages/Patient Number	Anomalies in Miscarriage Subgroup	LOV (mm)	ROV (mm)	PUV (mm)
Whole group (*n* = 518)	167	119/*n* = 79	24 (30.4%)	6.7 ± 2.0	5.9 ± 1.8	6.6 ± 1.4
P1 (*n* = 99)	27	M0/*n* = 91	24 (26.4%)	6.6 ± 1.8	5.4 ± 1.5	6.6 ± 1.6
		M1/*n* = 8	3 (37.5%)	5.5 ± 0.7 *	4.1 ± 1.2 *	5.7 ± 0.7 *
P2 (*n* = 171)	52	M0/*n* = 155	44 (28.4%)	7.2 ± 1.6	6.4 ± 1.7	6.9 ± 1.4
		M1/*n* = 12	5 (41.6%)	6.4 ± 1.7 *	5.8 ± 1.4	7.0 ± 2.1
		M2/*n* = 4	3 (75.0%)	5.4 ± 2.3 *	4.8 ± 1.8 *	5.8 ± 0.3 *
P3 (*n* = 78)	25	M0/*n* = 54	19 (35.2%)	7.8 ± 1.7	6.8 ± 1.7	7.4 ± 1.1
		M1/*n* = 21	5 (23.8%)	7.7 ± 1.8	6.3 ± 1.2	7.5 ± 1.1
		M2/*n* = 3	1 (33.3%)	6.5 ± 4.7 *	4.3 ± 0.3 *	6.0 ± 1.7 *
P4+ (*n* = 44)	20	M0/*n* = 13	13 (100.0%)	7.9 ± 1.8	7.2 ± 1.8	6.8 ± 1.4
		M1/*n* = 14	1 (7.1%)	8.1 ± 1.7	7.6 ± 2.2	7.2 ± 1.3
		M2/*n* = 8	2 (25.0%)	8.3 ± 2.1	7.0 ± 1.8	8.0 ± 1.4
		M3+/*n* = 9	4 (44.4%)	6.9 ± 2.5 *	6.4 ± 2.1 *	6.3 ± 1.0 *

The values shown in the table represent mean ±SD, as well as the number (percent) of cases within the respective group. Abbreviations: SD: standard deviation, LOV: left ovarian vein, ROV: right ovarian vein, and PUV: parauterine vein. The values marked with asterisks (*) were statistically significantly different compared to corresponding values in the respective subgroup without miscarriages (M0).

## Data Availability

Not applicable.
